# The effect of glycine administration on the characteristics of physiological systems in human adults: A systematic review

**DOI:** 10.1007/s11357-023-00970-8

**Published:** 2023-10-18

**Authors:** Janjira Soh, Shivaanishaa Raventhiran, Jasinda H. Lee, Zi Xiang Lim, Jorming Goh, Brian K. Kennedy, Andrea B. Maier

**Affiliations:** 1 Centre for Healthy Longevity, National University Health System (NUHS), Singapore, Singapore; 2 Healthy Longevity Translational Research Program, Yong Loo Lin School of Medicine, National University of Singapore (NUS), Singapore, Singapore; 3 Department of Biochemistry, Yong Loo Lin School of Medicine, National University of Singapore (NUS), Singapore, Singapore; 4 Department of Physiology, Yong Loo Lin School of Medicine, National University of Singapore (NUS), Singapore, Singapore; 5 Department of Medicine, Yong Loo Lin School of Medicine, National University of Singapore (NUS), Singapore, Singapore; 6 Department of Human Movement Sciences, @AgeAmsterdam, Amsterdam Movement Sciences, Faculty of Behavioural and Movement Sciences, Vrije Universiteit Amsterdam, Van Der Boechorstsraat 7, Amsterdam, 1081 BT The Netherlands

**Keywords:** Ageing, Geroprotector, Glycine, Healthspan, Lifespan, Physiological systems

## Abstract

Functional decline of physiological systems during ageing leads to age-related diseases. Dietary glycine increases healthy lifespan in model organisms and might decrease inflammation in humans, suggesting its geroprotective potential. This review summarises the evidence of glycine administration on the characteristics of eleven physiological systems in adult humans. Databases were searched using key search terms: ‘glycine’, ‘adult’, ‘supplementation’/ ‘administration’/ ‘ingestion’/ ‘treatment’. Glycine was administered to healthy and diseased populations (18 and 34 studies) for up to 14 days and 4 months, respectively. The nervous system demonstrated the most positive effects, including improved psychiatric symptoms from longer-term glycine administration in psychiatric populations. While longer-term glycine administration improved sleep in healthy populations, these studies had small sample sizes with a high risk of bias. Larger and long-term studies with more robust study designs in healthy populations to examine the effects of glycine administration on preventing, delaying or reversing the ageing process are warranted.

**Supplementary Information:**

The online version contains supplementary material available at 10.1007/s11357-023-00970-8.

## Introduction

Ageing is a complex biological phenomenon that occurs continuously in an organism with the passage of time; this results in a cumulative molecular damage and manifests as a progressive decline in organ functions of multiple physiological systems [1–4] leading to chronic diseases and disability [5, 6]. Therewith, optimising physiological function throughout the life course is critical to maximise an individual’s healthspan.

“Geroprotectors” are agents that enhance lifespan and healthspan in organisms by addressing the underlying cause of ageing and age-related diseases, thereby preventing, delaying, and/or reversing ageing processes [7]. The anti-cancer and anti-inflammatory effects of glycine have been observed in rodents [8–10]; and studies in humans suggest the potential of glycine supplementation to protect against metabolic diseases [8, 11], particularly by counteracting oxidative stress and inflammation [12, 13]. On the other hand, a chronic lack of glycine may impede growth, immune responses, and nutrient metabolism [14–16]. In animal models, glycine administration has been reported to extend the lifespan of *C. elegans* by up to 33% [17, 18], of rats by approximately 20% [19], and mice by 6% [20]. Given that glycine is inexpensive and likely safe for administration through oral supplementation, it is important to study its potential lifespan and healthspan enhancing properties as a geroprotector.

The physiological implications of glycine administration at the organ system level in human adults have not been comprehensively assessed. Hence, the aim of this systematic review is to summarise the effects of glycine administration on characteristics of physiological systems in adult humans.

## Methods

### Search strategy

This systematic review was registered with the International Prospective Register of Systematic Reviews (PROSPERO; CRD42022312730) and conducted according to the Preferred Reporting Items for Systematic Reviews and Meta-Analyses (PRISMA) guidelines. Four electronic databases were searched from date of inception to 29 April 2022: Embase, PubMed, Web of Science, and Cochrane Central Register of Controlled Trials. The search strategy was developed with the assistance of a senior tertiary librarian from the National University of Singapore, with expertise in research and search strategies. Search terms included but were not limited to: ‘glycine’, ‘adult’, ‘supplementation’/ ‘administration’/ ‘ingestion’/ ‘treatment’. Snowballing was used to search references within identified articles.

### Eligibility criteria

All study designs were considered. The inclusion criteria constituted the following: 1) population – adults (males and/or females) with a mean and/or median age of 18 years old and above; 2) intervention – administration of glycine in any combination of dose and medium through all reported routes (except topical administration), and independent of a placebo or control group were considered; 3) comparator(s)/control – glycine administration as the intervention compared with a placebo or no intervention, where applicable; 4) outcomes – characteristics of eleven physiological systems, which include the following systems: i) endocrine and metabolic, ii) nervous, iii) cardiovascular, iv) immune, v) digestive, vi) muscular, vii) renal, viii) reproductive, ix) integumentary, x) skeletal, and xi) respiratory.

Articles were excluded according to the following criteria: 1) animal and/or in vitro studies; 2) conference abstract, review, editorial, case reports, or letter to the editor; 3) studies investigating the following compounds: i) combined administration of glycine and another compound as an intervention, ii) precursors of glycine; glycine analogues; glycine derivatives; glycine (by-) products and intermediates; 4) studies investigating glycine administered topically, as a tracer compound, as an irrigation solution/fluid, and to solely measure pharmacokinetics and/or pharmacodynamics; 5) studies published in a non-English language; and 6) where full-text articles cannot be obtained.

### Article selection and data extraction

Two reviewers (JS, SR or JL) independently screened titles, abstracts, and full text articles for inclusion. Covidence systematic review software (Veritas Health Innovation, Melbourne, Australia) was used to screen the articles. Disagreements between reviewers were resolved by a third reviewer (JG). Data extraction was conducted independently by two authors (JS, SR or JL) and the following variables were extracted: author; year of publication; study design (e.g. randomised controlled trial, open label clinical trial, observational study, number of individuals per study arm, total sample size, dose and medium of glycine administration, intervention duration, and baseline conditions), population characteristics (age and sex); health status (e.g. healthy or diseased) and any reported changes in the characteristics of the aforementioned physiological systems.

### Data analysis

The outcomes of glycine administration were presented in a descriptive fashion in which a significant change was considered for outcomes reported with *p* < 0.05 in the following instances: i) pre-post outcomes of glycine administration, and ii) glycine administered as the intervention compound vs. placebo/control comparator(s) at the end of the intervention period. Extracted outcomes for each reported population was then stratified according to the eleven physiological systems and health condition (healthy or diseased). Whether these changes implied an overall positive or negative effect on the respective physiological system(s) was based on the significant change (*p* < 0.05) in at least one of the measured characteristics of a physiological system. Where both positive and negative effects had been reported for a physiological system, the overall effect of glycine administration on the physiological system was considered to have mixed effects. Where these changes did not imply (a) positive or negative effect(s) on a physiological system, these outcomes were considered inconclusive to the overall effect on the physiological system. Studies determining the short-term effect of a single bolus of glycine within a day and the longer-term effect over a period longer than a day were separated.

### Risk of bias

The risk of bias was assessed by two reviewers (JS, SR or JL). The revised Cochrane risk of bias tool for randomised trials [Cochrane risk of bias tool 2.0 (ROB 2)] was used to assess the risk of bias for randomised parallel-group [21] and crossover trials [22]. The tool used for randomised parallel group trials is based on five key sources of bias, namely: 1) the randomisation process, 2) deviations from intended intervention, 3) missing outcome data, 4) measurement of the outcome, and 5) selection of the reported result. For randomised crossover trials, the tool additionally includes bias arising from period and carryover effects. The risk of bias was categorised into “low risk”, “some concerns” and “high risk”. For non-randomised studies the Risk of Bias In Non-Randomised Studies – of Interventions (ROBINS-I) was employed [23]. This tool comprises seven key domains of bias: 1) confounding; 2) selection of participants; 3) classification of intervention; 4) deviation from interventions; 5) missing outcome data; 6) measurement of outcomes; and 7) selection of reported result overall. The risk of bias using ROBINS-I was rated accordingly: 0 – no information; 1 – low risk; 2 – moderate risk; 3 – serious risk; and 4 – critical risk.

## Results

### Study selection and characteristics of included studies

**Fig. 1:**
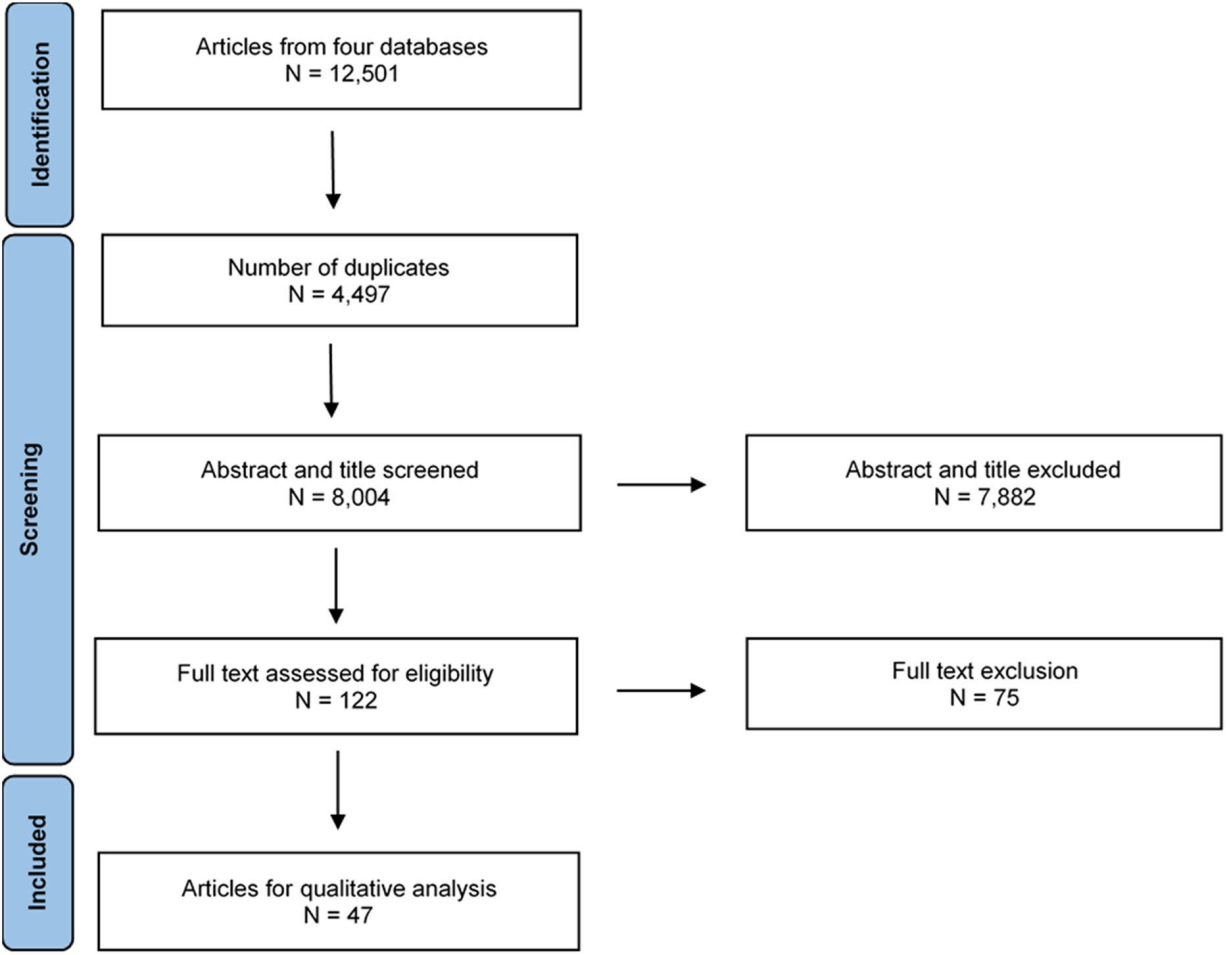
Schematic overview of article selection process

**Fig. 2:**
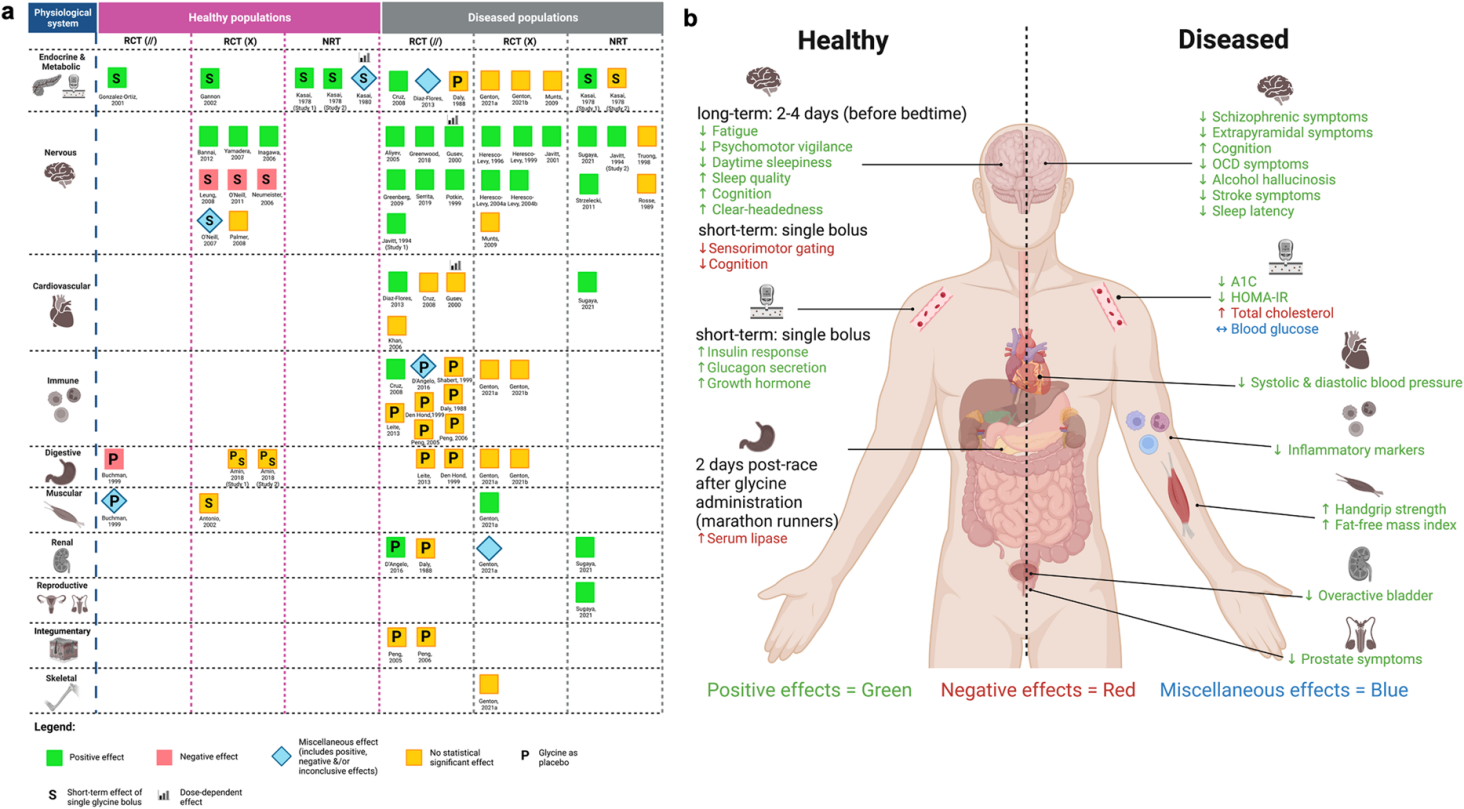
Summary of the effects of glycine administration in healthy and diseased populations. **A** Overall effects of administering glycine in healthy and diseased populations stratified by physiological systems and study types. **B** Summary of changes in characteristics on each physiological system in healthy and diseased populations with glycine administration. NRT, non-randomised trial; RCT (X), crossover randomised controlled trial; RCT (//), parallel-designed randomised controlled trial. *Created with BioRender.com*

The article selection process is presented in Fig. 1. After excluding 4,497 duplicates, 8,004 articles underwent title and abstract screening, of which 122 progressed to full-text screening and 47 articles describing 50 studies were included. Table 1 shows a comprehensive overview of the included articles. Most studies (42/50) were randomised controlled trials (RCT), of which half were parallel-group trials. The majority of studies (41/50) reported oral glycine ingestion as the mode of delivery. Eighteen out of 50 studies were in healthy populations, 34/50 in diseased populations, and 2/50 contained both healthy and diseased populations. The mean or median age ranged from 21.5 to 41.4 years for healthy populations and 29.5 to 67 years for diseased populations. Glycine was administered for a period of one day (single bolus) to 14 days in healthy populations and up to 4 months in diseased populations. Figure 2 and Supplementary Table A provide a summary of the effects of glycine administration in healthy and diseased populations stratified by physiological systems and study types.

**Table 1:** Articles describing glycine administration in humans stratified by characteristics of physiological systems measured

First author (year)	Population	Study type	Age (y)	Sex (%F)	Sample size (*n*)		Glycine duration, dose, delivery, medium	Comparator(s)
					Glycine Comparator(s)			
RCT (//)								
Endocrine & Metabolic system								
González-Ortiz (2001) [24]	Healthy first-degree relatives of T2DM patients	RCT (//)	Gly: 23.7(4.1) Comparator: 24.7(8.0)	67	6	6	Single bolus (short-term), 5 g (30 min before test), O, NR	Placebo (Magnesium oxide)
Endocrine & Metabolic + Cardiovascular systems								
Díaz-Flores (2013) [12]	Metabolic Syndrome	RCT (//)	Glycine*: 47.5 (8.2) Comparator*: 46.9 (7.8)	56*	30	30	3 m, 5 g X 3/d (15 g/d), O, glycine powder dissolved in water	Placebo (starch) dissolved in water
Endocrine & Metabolic + Cardiovascular + Immune systems								
Cruz (2008) [13]	T2DM	RCT(//)	58.5 (10)	54	38	36	3 m, 5 g X 3/d (15 g/d), O, powder dissolved in water	Placebo (starch powder dissolved in water)
Endocrine & Metabolic + Immune + Renal systems								
Daly (1988) [25]	GI malignancies	RCT (//)	Glycine: 62 (10) Comparator: 66 (8)	20	14	16	7d, 43 g X 1/d (43 g/d), E, L-glycine-supplemented enteral diet (solution)	L-arginine supplemented enteral diet (solution)
Nervous system								
Aliyev (2005) [26]	Alcohol hallucinosis	RCT (//)	Glycine: 42 (6.0) Comparator: 41.0 (5.0)	0	20	20	7d, 700 mg/d, S, glycine tablets	Placebo (NR)
Greenberg (2009) [27]	Obsessive compulsive disorder	RCT (//)	Glycine: 44.2 (14.3) Comparator: 36.1 (12.2)	63	12	12	12w, 30 g X 2/d (60 g/d), O, glycine powder dissolved in water or juice	Placebo (dextrose, fructose, fine granular citric acid, orange flavouring and ProSweet™ flavour enhancer dissolved in water or juice)
Greenwood (2018) [28]	Schizophrenia/ Schizoaffective disorder;	RCT (//)	37.8 (8.4)	57	12	10	6w, start @ 0.2 g/kg/d (0.2 g/kg X 2/d after 7d; 0.2 g/kg X 3/d after 14d onwards), O, NR	Placebo (NR)
Potkin (1999) [29]	Schizophrenia	RCT (//)	Glycine: 35.3 (5.26) Comparator: 34.4 (4.75)	12.5	12	12	12w, 10 g X 3/d after meals (30 g/d), O, solution of glycine dissolved in 1 oz water	Placebo (similar-tasting solution)
Javitt (1994) [30]	Schizophrenia	i) RCT (//) ii) Open label trial	i) Glycine: 36 (9.7) ii) Comparator: 38.1 (7.2)	i) 0 ii) NR	i) 7 ii) 15	i) 7 ii) NA	i) 8w, start at 2 g/d to maximum dose (0.4 g/kg bw – approx. 30 g/d) during first 2w, O, glycine powder dissolved in juice ii) 8w, 0.4 g/kg bw (approx. 30 g/d), O, glycine powder dissolved in juice	i) Placebo (taste-matched) ii) NA
Serrita (2019) [31]	Schizophrenia and alcohol dependence	RCT (//)	Glycine: 49.2 (4.84) Comparator: 48.6 (5.01)	0	10	10	12w, 0.8 g/kg, O, glycine powder mixed in solution	Placebo (powder mixed in solution)
Nervous + Cardiovascular systems								
Gusev (2000) [32]	Acute ischaemic stroke	RCT (//)	63.7 (10.1)*	45*	0.5 g/d: 53 1.0 g/d: 53 2.0 g/d: 53	53	5d, 0.5 or 1.0 or 2.0 g/d, S, tablet	Placebo tablet (similar in appearance & taste)
Cardiovascular system								
Khan (2006) [33]	Obstructive CAD	RCT (//)	61.1	21	111	112	6 m, 297.9 g X 2/d (595.8 g/d), O, glycine dissolved in water	Starch powder dissolved in water
Immune + Integumentary systems								
Peng (2006) [34]	Severe burn	RCT (//)	Patients: 36.5 (11.4); 18 – 60	40	23	25	14d, 0.5 g/kg/d, oral or tube feeding, granules	Glutamine granules (oral or tube feeding)
Immune + Renal systems								
D’Angelo (2016) [35]	Early preeclampsia	RCT (//)	Glycine*: 32.7 (4.8) Comparator: 31.1 (4.3)	100	20	20	Up to 7d, daily bolus of 60 ml injectable water containing 1% glycine, NR, solution	Antithrombin dissolved in 60 ml injectable water
Immune + Digestive systems								
Den Hond (1999) [36]	Crohn’s disease	RCT (//)	Glycine: 38.2 (13.4) Comparator: 25.0 (7.9)	All: 71 Glycine: 57 Comparator: 86	7	7	4w, 7 g X 3/d (21 g/d), O, glycine powder dissolved in water	Glutamine powder dissolved in water
Leite (2013) [37]	HIV/AIDS	RCT (//)	All: 37.3 (3.0) Glycine: 40.1 (1.9) Comparator: 34.2 (1.7)	22	24	22	10d, 25 g/d, O, 50 ml solution of orange juice enriched with glycine	Alanyl-glutamine in 50 ml solution of orange juice
Immune system								
Shabert (1999) [38]	HIV/AIDS	RCT (//)	Glycine*: 42; 33 – 53 Comparator*: 40; 30–50	All*: 10 Glycine*: 11 Compar-ator*: 8	9*	12*	12w, 40 g/d in 4 divided doses, NR, NR	L-glutamine + antioxidants (ascorbic acid, α-tocopherol, β-carotene, selenium, N-acetyl cysteine)
Digestive + Muscular systems								
Buchman (1999) [39]	Marathon runners	RCT (//)	25 – 49	All: 39 Gly: 20 Arg: 38	17	17	14d, 10 g X 3/d (30 g/d), O, glycine dissolved in water or orange juice	L-arginine dissolved in water or orange juice
Digestive system								
Manir (2014) [40]	Nonmetastatic pelvic malignancy	RCT (//)	Glycine*: 56.2 (9.6) Comparator*: 57.2 (8.1)	66	43*	42*	NR, NR (given 1 h prior radiation), O, NR	Glutamine granules dissolved in 100 ml fruit juice
Bushen (2004) [41]	HIV/AIDS	RCT (//)	All: 36 (6); Median: 36; 23 – 52	29	9	Glutamine: 11 Lo Ala-Gln: 11 Hi Ala-Gln: 10	7d, 46 g/d (spectrum), O, NR	i) Gln (30 g Gln + 15 g glycine/d) or, ii) Lo Ala-Gln (4 g Ala-Gln + 42 g glycine/d) or, iii) Hi Ala-Gln (44 g Ala-Gln/d)
Integumentary system								
Peng (2005) [42]	Severe burn	RCT (//)	36.5 (11.4); 18 – 60	40	23	25	14d, 0.5 g/kg/d, oral or tube feeding, granules	Glutamine granules (oral or tube feeding)
RCT (X)								
Endocrine & Metabolic system								
Gannon (2002) [43]	Healthy	RCT (X)	21 – 52	44	9	9	Single bolus (short-term) over 120 min, 1 mmol/kg lean bm, O	i) Water, or ii) 25 g glucose, or, iii) 1 mmol glycine/kg lean bm + 25 g glucose
Endocrine & Metabolic + Immune + Digestive systems								
Genton (2021b) [44]	Chronic haemodialysis with PEW	RCT (X)	Total*: 61.2 (13.7)	36*	37	37	4 m, 7 g X 2/d (14 g/d), O, granules	BCAA granules
Endocrine & Metabolic + Immune + Digestive + Muscular + Renal + Skeletal systems								
Genton (2021a) [45]	Chronic haemodialysis with PEW	RCT (X)	Total*: 61.2 (13.7) BCAA-Glycine*: 63.3 (13.4) Glycine-BCAA*: 58.6 (14.2)	36*	37	37	4 m, 7 g X 2/d (14 g/d), O, granules	BCAA granules
Endocrine & Metabolic + Nervous systems								
Munts (2009) [46]	CRPS with dystonia	RCT (X)	41{34 – 51}	95	19	19	4w, 21 mg/ml, IT, solution	Placebo (0.9% sodium chloride IT solution)
Nervous system								
Bannai (2012) [47]	Healthy	RCT (X)	41.4; 30 – 61	0	10	10	3 consecutive nights, 3 g/d (30 min before bedtime), O, flavoured glycine	Flavoured placebo (reduced form of malt sugar)
O’Neill (2011) [48]	Healthy	RCT (X)	23 (4.1); 18 – 45	0	16	16	Single bolus (short-term), 0.8 g/kg, O, glycine mixed with 200 ml orange juice	Placebo (flour powder mixed with 200 ml orange juice to mimic texture of glycine treatment)
Palmer (2008) [49]	Healthy	RCT (X)	23.15 (4.26); 19 – 36	0	13	13	Single bolus (short-term), 0.8 g/kg, O, glycine powder dissolved in 200 ml orange juice	Placebo (flour powder mixed with 200 ml orange juice)
Leung (2007) [50]	Healthy	RCT (X)	23 (4.1); 19 – 36	0	16	16	Single bolus (short-term), 0.8 g/kg bw, O, mixed with 200 ml orange juice	Flour mixed with 200 ml orange juice
O’Neill (2007) [27]	Healthy	RCT (X)	23 (4.1); 19 – 36	0	16	16	Single bolus (short-term) over 90 min, 0.8 g/kg bw, O, glycine powder mixed with 200 ml orange juice	Placebo (flour powder mixed with 200 ml orange juice)
Yamadera (2007) [51]	Healthy	RCT (X)	40.5 (10.1); 30 – 57	73	11	11	2 consecutive nights, 3 g/d (1 h before bedtime), O, flavoured glycine	Flavoured placebo (reduced form of malt sugar)
Neumeister (2006) [52]	Healthy	RCT (X)	28.5 (10.5)	33*	13	13	Single bolus (short-term), 200 mg/kg bw over 45 min, IV infusion	Placebo (saline solution)
Inagawa (2006) [53]	Dissatisfaction with sleep	RCT (X)	31.1; 24 – 53	100	19	0	4d, 3 g/d (1 h before bedtime), O, flavoured glycine (medium NR)	Placebo (flavoured)
Heresco-Levy (2004b) [54]	Schizophrenia	RCT (X)	44.7 (10.8)	24	17	17	6w, initiated at 4 g/d (↑ 4 g/d until 0.8 g/kg bw/d after 10d – 17d) in 3 divided doses, O, glycine powder dissolved in water (20% solution)	Placebo (glucose solution)
Heresco-Levy (2004a) [55]	Schizophrenia	RCT (X)	42.4	41	17	17	6w, start at 4 g/d (↑ 4 g/d until 0.8 g/kg bw/d) in 3 divided doses, O, glycine powder dissolved in water	Placebo (glucose solution)
Javitt (2001) [56]	Schizophrenia	RCT (X)	39.6 (5.5)	33	12	12	6w, 0.8 g/kg/d, O, glycine powder dissolved in orange juice	Placebo (glucose) dissolved in orange juice
Heresco-Levy (1999) [57]	Schizophrenia	RCT (X)	38.8 (11.0)	45	22	22	6w, initiated at 4 g/d (↑ 4 g/d until 0.8 g/kg bw/d after 9d – 19d) in 3 divided doses, O, glycine powder dissolved in water (20% solution)	Placebo (glucose solution)
Heresco-Levy (1996) [58]	Schizophrenia	RCT (X)	41.4* 22 – 60*	55*	12	12	6w, start at 4 g/d (↑ 4 g/d until 0.8 g/kg bw/d) in 3 divided doses, O, glycine powder dissolved in water	Placebo (glucose solution)
Digestive system								
Amin (2018) [59]	i) Healthy ii) Healthy	i) RCT (X) ii) RCT (X)	i) 39.4 (11.4) ii) 36.0 (10.8)	i) 86 ii) 89	i) 7 ii) 9	i) 7 ii) 9	For both studies: Single bolus (short-term), 17.1 mmol, O, hypromellose capsules	For both studies: i) L-arginine hydrocholoride in hypromellose capsules or, ii) empty hypromellose capsules (vehicle)
Luiking (1998) [60]	Healthy	RCT (X)	24.2 (4.1)	0	10	10	8d, 13 g/d over 4 doses, O, solution	Placebo (glucose + chloride) solution
Muscular system								
Antonio (2002) [61]	Resistance-trained	RCT (X)	21.5 (0.3)	NR	6	6	Single bolus (short-term), 0.3 g/kg, O, glycine mixed with 250 ml calorie-free fruit juice	i) Glutamine mixed with calorie-free fruit juice, or ii) Placebo (calorie-free fruit juice only)
Non-randomised trials								
Endocrine & Metabolic systems								
Kasai (1980) [62]	Non-obese normal	Open-label trial (//)	18 – 46	48	25	NA	Single bolus (short-term) over 150 min, 4 g or 8 g, IV; single bolus (short-term) over 180 min, 12 g, IV	NA
Kasai (1978) [63]	i) Non-obese normal; gastroduodenal anastomosis (partially gastrectomied) ii) Non-obese normal; non-obese diabetics	i) Open-label trial ii) Open-label trial	i) 20 – 70 ii) 20 – 70	i) 39 ii) 47	i) 31 ii) 15	i) NA ii) NA	i) Single bolus (short-term) over 180 min, 250 ml 0.3 M, O ii) Single bolus (short-term) over 180 min, 250 ml 0.3 M, ID	i) NA ii) NA
Nervous + Cardiovascular + Renal + Reproductive systems								
Sugaya (2021) [64]	Overactive bladder	Pilot (//)	67 (16)	20	20	20	4w, 3 g X 2/d (6 g/d), O, NR	Placebo (glucose)
Nervous system								
Truong (1988) [65]	Myoclonus	Open-label trial; crossov-er trial	38; 18 – 58	NR	7	NA	Up to 9w, start @ 200 mg X 3/d (600 mg/d) then ↑ daily by 300 mg until therapeutic effect achieved or up to max dose 6 g/d in 3 divided doses over 6w, O, capsule	NA
Strzelecki (2011) [66]	Schizophrenia	Open-label trial	32.3 (8.8)*; Median: 29.5; 20 – 50*	45*	32	NA	6w, 0.8 g/kg bm/24 h/3 doses, O, glycine crystillizate dissolution in approx. ½ glass of water or orange juice	NA
Javitt (1994) [30]	Schizophrenia	i) RCT (//) ii) Open label trial	i) Glycine: 36 (9.7) ii) Comparator: 38.1 (7.2)	i) 0 ii) NR	i) 7 ii) 15	i) 7 ii) NA	i) 8w, start at 2 g/d to maximum dose (0.4 g/kg bw – approx. 30 g/d) during first 2w, O, glycine powder dissolved in juice ii) 8w, 0.4 g/kg bw (approx. 30 g/d), O, glycine powder dissolved in juice	i) Placebo (taste-matched) ii) NA
Rosse (1989) [67]	Chronic psychotic disorder	Open-label trial	38; 30 – 68	0	6	NA	4d – 8w, 10.8 g in 3 divided doses daily, O, glycine capsule	NA

Age data are presented as: mean (SD); or median {IQR}

*(X)* crossover trial, *(//)* parallel trial, (*) value(s) given only for participants who completed trial and/or included in analysis, *AIDS* acquired immune deficiency syndrome, *Arg* arginine, *BCAA* branched-chain amino acid, *bm* body mass, *bw* body weight, *d* day, *CAD* coronary artery disease, *DCS* D-cycloserine, *E* enteral, *F* female, *g* gram, *GI* gastrointestinal, *Gly* glycine, *Hi* high dose, *HIV* human immunodeficiency virus, *I* inhalation, *ID* intraduodenal, *IT* intrathecal, *IV* intravenous, *Lo* Low dose, *O* oral ingestion, *PEW* protein energy wasting, *NA* not applicable, *NR* not reported, *m* month, *RCT* randomised controlled trial, *T2DM* type 2 diabetes mellitus, *w* week, *y* years

### Endocrine & metabolic systems

In healthy populations, 5/5 studies reported changes in endocrine and metabolic system where a single oral glycine bolus improved insulin responses [24, 43], and increased circulating concentrations of glucagon [43] and growth hormone [63]. In a non-obese healthy population, an inconclusive dose-dependent outcome was observed when a single intravenous (IV) administration of 4 g glycine increased serum growth hormone concentrations, while 12 g glycine increased serum blood sugar levels [62]. In diseased populations, oral glycine administration of 5 g X 3/day over 3 months showed positive effects in type 2 diabetes mellitus (T2DM) patients, including decreased glycosylated haemoglobin (A1C) (%), Homeostatic Model Assessment for Insulin Resistance (HOMA-IR) and fasting blood glucose [13]; while the same dose over 3 months decreased A1C (%), but increased fasting blood glucose and total cholesterol levels when compared to baseline in metabolic syndrome (MetS) patients [12]. The latter study also reported miscellaneous effects that differed between males and females when glycine was compared to placebo administration instead, including: i) increased blood levels of glucose and high-density lipoprotein (HDL) in females; and ii) increased blood levels of total cholesterol, HDL, and systolic blood pressure; and decreased blood levels low-density lipoprotein and A1C (%) [12]. In gastroduodenal anastomosis patients, 0.3 M of a single oral or intraduodenal glycine bolus administration also increased circulating growth hormone concentrations [63] (Table 1, Fig. 2, Supplementary Table A).

### Nervous system

The nervous system was examined in healthy populations in eight studies [47–53, 68]. Improved sleep quality, alertness and cognition, and decreased fatigue and sleepiness was observed in three populations receiving 3 g/day oral administration of glycine 30 min – 1 h before bedtime over 2 – 4 days [47, 51, 53]. Higher single bolus of 0.8 g/kg body weight orally or 200 mg/kg body weight intravenously showed negative effects on sensorimotor gating and cognitive performance in healthy populations [48, 50, 52]. Of the eight studies in healthy populations, one reported inconclusive effects with glycine administration [68], while another reported a statistically insignificant effect [49]. In diseased populations, 15/18 studies reported significant positive effects with glycine administration [26–32, 54–58, 64, 66], especially in psychiatric populations where oral glycine administration of 0.2 – 0.8 g/kg body weight daily over 6 – 12 weeks improved schizophrenic [28–31, 54–58, 66]/psychiatric symptoms, extrapyramidal symptoms and cognition. In overactive bladder patients, sleep latency decreased with 3 g X 2/day of oral glycine over 4 weeks [64] (Table 1, Fig. 2, Supplementary Table A).

### Cardiovascular system

The cardiovascular system was not assessed in healthy populations. In diseased populations, positive effects included decreased systolic blood pressure with an oral glycine dose of 5 g X 3/day over 3 months in MetS patients [12], and with an oral glycine dose of 3 g X 2/day over 4 weeks in overactive bladder patients [64] (Table 1, Fig. 2, Supplementary Table A).

### Immune system

The immune system was not assessed in healthy populations. In T2DM patients, significant positive immune system effects were observed after 3 months of 5 g X 3/day oral glycine ingestion, including decreased proinflammatory cytokines such as interleukin-6 (IL-6), interferon-gamma (IFN-γ), tumour necrosis factor- receptor 1 (TNF-RI), resistin, and interleukin-1 beta (IL-1β) [13] (Table 1, Fig. 2, Supplementary Table A).

### Digestive system

In a healthy population of marathon runners, 10 g X 3/day oral glycine over 14 days prior to a marathon run showed significantly higher post-race serum lipase concentrations compared to baseline, suggesting mildly ameliorated pancreatic injury [39] (Table 1, Fig. 2, Supplementary Table A).

### Muscular system

In a healthy population of marathon runners, 10 g X 3/day oral glycine over 14 days prior to a marathon run showed significant pre-post effects with increased 2 days post-race serum creatinine phosphokinase (CPK) concentrations (used as a surrogate marker for muscle injury) [39]. However, this rise in serum CPK concentrations was attributed to skeletal muscle trauma induced from marathon running; and it was concluded that glycine administration was not useful in preventing skeletal muscle injury [39]. No significant effect on upper and lower body strength was reported in resistance-trained adults with a single oral glycine bolus of 0.3 g/kg body weight 1 h prior to assessment [61]. Patients undergoing chronic haemodialysis with protein energy wasting (PEW) showed positive effects, including improvements in handgrip strength and fat-free mass index following oral glycine administration of 7 g X 2/day over 4 months [45] (Table 1, Fig. 2, Supplementary Table A).

### Renal system

The renal system was not assessed in healthy populations. In diseased populations, positive effects included mitigation of symptoms in patients with overactive bladder administered with 3 g X 2/day of oral glycine over 4 weeks; and decreased daily proteinuria in early preeclampsia patients administered with placebo 1% glycine solution for up to 7 days [35]. Although increased levels of pre-dialysis urea and normalised protein catabolic rate (nPCR) were reported in chronic haemodialysis patients with PEW following oral glycine administration of 7 g X 2/day over 4 months, these results were attributed to the patients’ compliance to glycine treatment [45] (Table 1, Fig. 2, Supplementary Table A).

### Reproductive system

The reproductive system was not assessed in healthy populations. In a diseased population with overactive bladder [64], positive effects on prostate symptoms, including nocturia and urinary urgency, were reported at a dose of 3 g X 2/day over 4 weeks [64] (Table 1, Fig. 2, Supplementary Table A).

### Integumentary system

The integumentary system was not assessed in healthy populations. In diseased populations, both studies assessed the integumentary system in the same population with severe burn [34, 42]. No significant on the area and depth of burns was observed with oral glycine of 0.5 g/kg/day over 14 days [34, 42] (Table 1, Fig. 2, Supplementary Table A).

### Skeletal system

The skeletal system was not assessed in healthy populations. Bone mineral density did not change in chronic haemodialysis patients with PEW given 7 g × 2/day of oral glycine administration over 4 months compared to baseline [45] (Table 1, Fig. 2, Supplementary Table A).

### Respiratory system

The respiratory system was not assessed in healthy or diseased populations.

### Risk of bias across studies

**Fig. 3:**
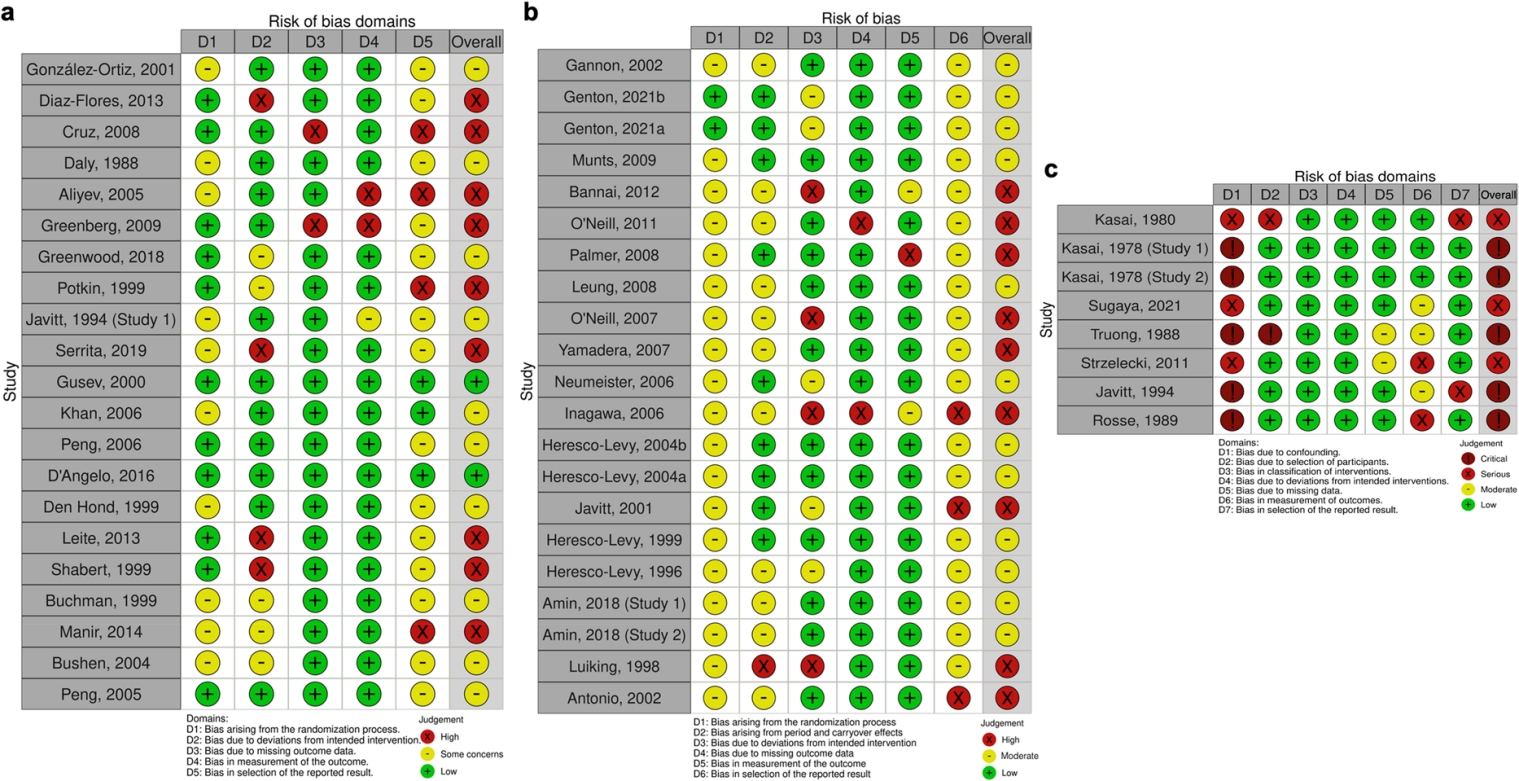
Overview of risk of bias based on the Cochrane risk of bias for included human studies. **a** ROB2 for RCT parallel group trials. **b** ROB2 for RCT crossover trials. **c** ROBINS-I for non-randomised trials

Figure 3a presents the Cochrane risk of bias ratings for parallel-designed RCTs. The majority of the studies were classified as either some concerns or high for overall risk of bias; with 10/21 and 9/21 of parallel-group RCTs, respectively; with the remaining 2/21 studies classified as having a “low” overall risk of bias [32, 35]. Figure 3b shows the Cochrane risk of bias ratings for crossover-designed RCTs. These studies were classified as either “some concerns” or “high” overall risk, by comprising of 12/21 and 9/21 of crossover-designed RCTs, respectively. Most of these studies were rated as “some concerns” for the domains “bias arising from the randomisation process” and “bias in selection of the reported result”; hence, none of the crossover studies was classified as having an overall “low” risk of bias. Figure 3c shows the Cochrane risk of bias ratings for non-randomised studies of interventions. The majority of these studies were open-label studies, and were either classified as “critical” (5/8) or “serious” (3/8) overall of bias. This result was attributed to all of these studies being rated as “critical” or “serious” risk for the domain “bias due to confounding”.

A meta-analysis combining the extracted data to ascertain the overall effect of glycine administration on the characteristics for each physiological system could not be performed due to the large heterogeneity and nature of reported outcomes and statistical presentation of the data.

## Discussion

Glycine administration may improve the characteristics of multiple physiological systems, but there is limited evidence supporting their preventative effect for healthy populations. The majority of the physiological systems demonstrated significant positive effects on glycine that were mostly related to the nervous system with longer-term glycine administration, especially in diseased populations afflicted with psychiatric illnesses such as Schizophrenia. The positive effects reported on healthy populations included improved sleep and decreased daytime fatigue [47, 51, 53] and improved insulin responses [24, 43]. On the other hand, negative effects were mainly reported in studies giving a higher glycine dose in a single bolus [48, 50, 52]. This disparity in outcomes in studies on healthy populations may be attributed to variation in dosages and intervention periods.

Nutritional studies have highlighted that the amount of glycine available in humans and animals is inadequate to satisfy metabolic requirements, suggesting the need for dietary glycine supplementation [8, 14, 69, 70]. Several lines of evidence support the hypothesis of accelerated ageing in this psychiatric disorder which reduces the average lifespan of patients by 15 to 20 years compared with the general population [71, 72]. Besides increased mortality risk, Schizophrenia shares risk factors with other age-related conditions such as cognitive decline, metabolic abnormalities, and cardiovascular ageing [73]. However, the included studies on Schizophrenic populations in the present review have thus far only measured the effects of glycine administration on the nervous system, raising the issue of whether benefits may be observable in other physiological systems in this context.

Improved psychiatric symptoms in populations afflicted with psychiatric diseases, were accompanied by improved cognition [57, 58, 66] and extrapyramidal symptoms [54]. Moreover, in chronic haemodialysis patients with PEW, improvements were observed in handgrip strength and fat-free mass index [45]. Considering the involvement of the skeletal muscle in movement, together, it is plausible that these effects may, in turn, positively influence a host of functions associated with multiple physiological systems. Patients with sarcopenia, often have cognitive impairment associated with a decline in muscle strength, mass and function [74], along with other metabolic conditions such as diabetes mellitus [75] and metabolic syndrome [76].

Schizophrenia is hypothesised to result from the hypofunctioning of NMDA receptors [77]. Several reports cited herein have particularly underscored the potential effect of glycine on the N-methyl-D-aspartate (NMDA) receptor in eliciting positive neurological outcomes. Consistent with this notion, brain ageing is associated with the reduced NMDA receptor function, in turn leading to declined memory and learning performance [78]. Stimulation of glycine binding to NMDA receptors have been shown to ameliorate extrapyramidal symptoms of neuromuscular function [79] and a study has shown that glycine administration could improve extrapyramidal and cognitive symptoms in Schizophrenic patients [54]. NMDA receptors have also been implicated in other age-related diseases such as diabetes [80] and hypertension [81]. In healthy populations, oral glycine administration before bedtime has been shown to improve sleep quality through the action of glycine on NMDA receptors in the suprachiasmatic nucleus (SCN), the master circadian pacemaker, by promoting hypothermia and vasodilation [47]. Therefore, one possible mechanism by which glycine may confer its geroprotective effects may be through its action on NMDA receptors; although further research is required to understand the chronotherapeutic and tissue-specific effects of such an interaction and the interplay between multiple physiological systems in healthy and diseased populations.

### Strength and limitations

This systematic review is the first to evaluate the effects of glycine on multiple physiological systems in adult humans; and is key in informing and substantiating health claims related to glycine.

In assessing the risk of bias across studies, the two studies classified as having a low overall risk of bias were parallel-group RCTs on diseased populations [32, 35], with none on healthy populations. Thus, conclusions drawn from the effect of glycine administration on the physiological systems should generally be treated judiciously, particularly for studies on healthy populations in improving in improving sleep quality, fatigue and alertness [47, 51, 53] where the evidence stem from studies of small sample sizes with overall high risk of bias. The search strategy was designed to be broad and inclusive, since articles on the topical administration of glycine such as its application on the skin have been excluded, this may account for the low number of studies on the integumentary system. Publication bias may have skewed analysis toward positive findings. Hence, the conclusions of this systematic review warrant judicious consideration. Formal statistical analysis was not conducted and results are interpreted on reported p-values, which is dependent on the sample size of the studies.

## Conclusions

Glycine administration is most effective in improving characteristics of the nervous system, especially in ameliorating neurological symptoms in populations with psychiatric illnesses, most notably in Schizophrenia. Ageing is associated with the decline in function of various physiological systems and elucidating the molecular underpinnings and mechanisms of these disease states are critical in determining strategies to prevent ageing and age-related diseases. Although the administration of glycine may improve the characteristics of multiple physiological systems, there is currently limited evidence supporting their preventative effect for healthy populations, which warrants the need for future research. Importantly, larger and more robustly-designed RCTs are necessary to strengthen the current evidence on the potential of glycine administration in conferring benefits in adult humans. It would be prudent to conduct more studies on healthy populations to particularly establish the optimum dosage, route and medium of delivery, intervention duration, and timing of glycine administration for optimal organ function over multiple physiological systems to prevent the onset of age-related diseases, or to delay and potentially reverse the ageing process. Notwithstanding, the evidence to-date may suggest a simple and effective preventative strategy to enhance healthspan through oral glycine supplementation. Considering the pleiotrophic effect of glycine on multiple physiological systems demonstrated in this review, future studies should assess the effects of glycine administration on a diverse range of physiological systems in both healthy and diseased populations; and potentially, the differences in these outcomes between males and females.

### Supplementary Information

Below is the link to the electronic supplementary material.

Supplementary file1 (PDF 7 KB)

Supplementary file2 (DOCX 68 KB)

Supplementary file3 (PDF 31 KB)
