# Supplementary material for: The effect of glycine administration on the characteristics of physiological systems in human adults: A systematic review
Source: GeroScience. 2023 Oct 18;46(1):219–39. doi: 10.1007/s11357-023-00970-8 (PMC10828290; doi:10.1007/s11357-023-00970-8)
Supplement: Supplementary file 1 — Supplementary file1 (PDF 7 KB) [file 11357_2023_970_MOESM1_ESM.pdf]

Supplementary Information A – Search strategy

The following search query was executed in Pubmed, EMBASE, Cochrane (CENTRAL), and Web of Science:

(supplement* OR treat* OR administrat* OR administer* OR ingest*)

AND

(men OR man OR adult OR women OR woman OR older OR elderly OR middle age* OR old OR adult* OR male* OR female* OR individual*)

AND

glycine
