# Supplementary material for: The effect of glycine administration on the characteristics of physiological systems in human adults: A systematic review
Source: GeroScience. 2023 Oct 18;46(1):219–39. doi: 10.1007/s11357-023-00970-8 (PMC10828290; doi:10.1007/s11357-023-00970-8)
Supplement: Supplementary file 2 — Supplementary file2 (DOCX 68 KB) [file 11357_2023_970_MOESM2_ESM.docx]

**Supplementary Table A. Changes in physiological characteristic(s) of glycine administration when compared with baseline or placebo in humans stratified by physiological systems.**

| First author (year) | Population | Design | Physiological characteristic(s) measured | Glycine vs. Placebo | Pre-post Glycine | Outcomes | | | | | | | |
| --- | --- | --- | --- | --- | --- | --- | --- | --- | --- | --- | --- | --- | --- |
| **RCT (//)** | | | | | | | | | | | | | |
| **Endocrine & Metabolic system** | | | | | | | | | | | | | |
| Gonzalez-Ortiz (2001) [25] | Healthy first-degree relatives of T2DM patients | RCT (double-blind, placebo) | - Insulin response & action - Glucose metabolism | ↑Insulin response | NS (for all outcome measures) | E&M | | | | | | | |
| **Endocrine & Metabolic + Cardiovascular systems** | | | | | |  | | | | | | | |
| Diaz-Flores (2013) [12] | Metabolic Syndrome | RCT (double-blind, placebo) | - Glucose - TC, LDL, HDL, TG - A1C - Insulin - SBP - DBP | ↑Glucose: ♀  ↑TC: **♂**  ↓LDL: ♀  ↑HDL: **♂,** ♀  ↓A1C: **♂**  ↑SBP: **♂**  DBP: NR  Insulin: NR  TG: NR | ↑Glucose  ↑TC  ↓A1C  ↓SBP  ↔LDL  ↔HDL  ↔DBP  ↔Insulin  TG: NR | E&M | | | C | | | | |
| **Endocrine & Metabolic + Cardiovascular + Immune systems** | | | | | | | | | | | | | |
| Cruz  (2008) [13] | T2DM | RCT (double-blind, placebo) | - Fasting blood glucose - TC, LDL, HDL, TG - A1C - Fasting insulin - Insulin resistance (HOMA-IR) - SBP - DBP - IL-1β - IL-6 - TNF-RI - IFN-γ - Resistin | ↓TNF-RI  ↑IFN-γ  ↓AIC  ↔HOMA-IR  ↔SBP  ↔DBP  ↔TC, LDL, HDL, TG  ↔Fasting insulin  ↔Fasting blood glucose | ↓HOMA-IR  ↓AIC  ↓Fasting blood glucose  ↓TNF-RI  ↑IFN-γ  ↔Fasting insulin  TC, LDL, HDL, TG: NR  SBP: NR  DBP: NR  IL-6: NR  IL-1β: NR  Resistin: NR | E&M | | | C | | | I | |
| **Endocrine & Metabolic + Immune + Renal systems** | | | | | | | | | | | | | |
| Daly (1988) [69] | GI malignancies | RCT (double-blind, placebo*) | - Plasma insulin - Plasma glucagon - Serum cortisol - Serum growth hormone - Serum somatomedin C (IGF1) - Caloric intake - Nitrogen intake - Nitrogen balance - Serum cortisol - Serum growth hormone - Serum glucose - Serum creatinine - Blood urea nitrogen - CD2 - CD4 - CD8 - Antigen-presenting monocytes - Stimulation index | NA | NS (for all outcome measured) | E&M | | | I | | R | | |
| **Nervous system** | | | | | | | | | | | | | |
| Aliyev (2005) [36] | Alcohol hallucinosis | RCT (double-blind, placebo) | Clinical symptoms (overall symptoms score) of acute alcohol hallucinosis:   - verbal hallucinations - hallucinatory delusions - affective frustration | ↓Overall symptoms score | NR | N | | | | | | | |
| Greenberg (2009) [37] | Obsessive compulsive disorder | RCT (double-blind, placebo) | - Y-BOCS, total - Y-BOCS, OBS subscale - Y-BOCS, COM subscale | ↓Y-BOCS total  ↓Y-BOCS OBS subscale  ↔Y-BOCS COM subscale | NR | N | | | | | | | |
| Greenwood (2018) [38] | Schizophrenia;  Schizoaffective disorder; | RCT (double-blind, placebo) | - PANSS (total score; negative score, positive score, general subscale) - CDRS - WSAS - WTAR - MMN | PANSS (↓total score; ↓negative score, ↓general subscale, ↔positive score)  ↔CDRS  ↔WSAS  ↔WTAR  ↔MMN | NS (for all outcome measures) | N | | | | | | | |
| Potkin (1999) [41] | Schizophrenia | RCT (double-blind, placebo) | - BPRS - SANS - SAS | ↑BPRS  ↔SANS  ↔SAS | NR | N | | | | | | | |
| Javitt (1994) [42] | Schizophrenia | RCT (double-blind, placebo) | - PANSS (negative symptoms, positive symptoms, general psychopathology) | PANSS (↓negative symptoms, ↔positive symptoms, ↔general psychopathology) | PANSS (↓negative symptoms, ↔positive symptoms, ↔general psychopathology) | N | | | | | | | |
| Serrita (2019) [40] | Schizophrenia and alcohol dependence | RCT (double-blind, placebo) | - Drinking (drinks/week; drinking days; heavy drinking days; drinks/day; % heavy drinking days; % no. of drinking days) - Craving (OCDS: total score; obsessions; compulsions) - PANSS (negative; positive; general symptoms) | NA | ↓drinking (drinks/week; drinking days; heavy drinking days; drinks/day; % heavy drinking days; % no. of drinking days)  ↓Craving (OCDS: total score; obsessions; compulsion)  PANSS (↓positive symptoms, ↔negative symptoms, ↔positive symptoms, ↔general psychopathology) general symptoms) | N | | | | | | | |
| **Nervous + Cardiovascular systems** | | | | | | | | | | | | | |
| Gusev  (2000) [39] | Acute ischaemic stroke | RCT (double-blind, placebo) | - - OSS   - SSS   - blood serum AutoAB to glutamate NMDA receptors   - CSF glutamate   - CSF glycine   - CSF aspartate   - CSF GABA   - CSF TBARS - blood pressure - heart rate - ECG | For 1g/day and 2g/day:  ↑OSS  ↑SSS  ↓AutoAB  ↓CSF glutamate  ↓CSF aspartate  ↓CSF GABA  ↓CSF TBARS  ↔CSF glycine  ↔blood pressure  ↔heart rate  ↔ECG | NR | N | | C | | | | | |
| **Cardiovascular system** | | | | | | | | | | | | | |
| Khan  (2006) [70] | Obstructive CAD | RCT (double-blind, placebo) | - Target LAD, LCX, LM, RCA - Lesion type A, B1, C - Target vessel occluded (subtype C) - Obstruction length - Minimal luminal diameter - Stenosis (%) - Restenosis rate | ↓ LCX  ↑ RCA  ↔LAD, LM  ↔Lesion type A, B1, C  ↔Target vessel occluded (subtype C)  ↔Obstruction length  ↔Minimal luminal diameter  ↔Stenosis (%)  ↔Restenosis rate | NR | C | | | | | | | |
| **Immune + Integumentary systems** | | | | | | | | | | | | | |
| Peng  (2006) [55] | Severe burn | RCT (double-blind, placebo*) | - LTR - NPI - CD4+/CD8+ - IL-2 - IgA - IgM - IgG - C3 - C4 - Area & depth of burns (TBSA; full thickness burns area) | NA | NS (for all outcome measures) | I | | Int | | | | | |
| **Immune + Renal systems** | | | | | | | | | | | | | |
| D’Angelo  (2016) [53] | Early preeclampsia | RCT (double-blind, placebo*) | - White blood cell & platelet count - Prothrombin time - Activated partial thromboplastin time ratios - p-fibrinogen - RBC - Antithrombin - D-dimer - Protein C - Protein S - Soluble thrombomodulin - Thrombin-antithrombin complex - TNF-α - IL-6 - PAI-2 - Activated protein C - daily proteinuria - serum creatinine - Serum uric acid | NA | ↑ fibrinogen  ↑ Protein C  ↑ d-dimer  ↑ d-dimer  ↑ IL-6  ↓ PAI-2  ↓ daily proteinuria  ↔white blood cell & platelet count  ↔prothrombin time  ↔activated partial thromboplastin time ratios  ↔RBC  ↔antithrombin  ↔Protein S  ↔soluble thrombomodulin  ↔thrombin-antithrombin complex  ↔TNF-α  ↔Activated protein C  ↔serum creatinine  ↔Serum uric acid | I | | R | | | | | |
| **Immune + Digestive systems** | | | | | | | | | | | | | |
| Den Hond  (1999) [71] | Crohn’s disease | RCT (double-blind, placebo*) | - CRP - Cr-EDTA - CDAI | NA | NS (for all outcome measures) | I | | D | | | | | |
| Leite  (2013) [72] | HIV/AIDS | RCT (double-blind, placebo*) | - CD4 - HIV viral load - % lactulose excretion - % mannitol excretion - Lactulose/mannitol ratio | NA | NS (for all outcome measures) | I | | D | | | | | |
| **Immune system** | | | | | | | | | | | | | |
| Shabert  (1999) [73] | HIV/AIDS | RCT (double-blind, placebo*) | - CD4+ T lymphocytes | NA | NS (for all outcome measures) | I | | | | | | | |
| **Digestive + Muscular systems** | | | | | | | | | | | | | |
| Buchman  (1999) [50] | Marathon runners | RCT (double-blind, placebo*) | - Intestinal permeability (urinary lactulose: mannitol concentration after oral lactulose & mannitol) - Heme positive stool - Pancreatitis (serum amylase & lipase) - Nausea & vomiting - Belching & indigestion - Abdominal pain & bloating - Diarrhoea - Extremity (muscle) pain - Muscle injury (serum CPK) | NA | ↑Serum lipase  ↑Serum CPK  ↔Intestinal permeability  ↔Heme positive stool  ↔Pancreatitis (serum amylase & lipase)  ↔Nausea & vomiting  ↔Belching & indigestion  ↔Abdominal pain & bloating  ↔Diarrhoea  ↔Extremity (muscle) pain | D | | M | | | | | |
| **Digestive system** | | | | | | | | | | | | | |
| Manir  (2014) [74] | Nonmetastatic pelvic malignancy | RCT (double-blind, placebo*) | - Nausea - Vomiting - Enteritis - Diarrhoea - Distension/abdominal bloating - Proctitis | NA | NR | NA | | | | | | | |
| Bushen  (2004) [75] | HIV/AIDS | RCT (double-blind, placebo*) | - Intestinal permeability (lactulose and mannitol excretion levels) - Stool samples (ova and parasites) - Frequency of diarrhoea - Stool consistency - ARV drug absorption - Other GI complaints (vomiting; anorexia; abdominal cramping) | NA | NR | NA | | | | | | | |
| **Integumentary system** | | | | | | | | | | | | | |
| Peng  (2005) [54] | Severe burn | RCT (double-blind, placebo*) | - Area & depth of burns (TBSA; full thickness burns area) | NA | NS (for all outcome measures) | I | | | | | | | |
| **RCT (X)** | | | | | | | | | | | | | |
| **Endocrine & Metabolic system** | | | | | | | | | | | | | |
| Gannon  (2002) [24] | Healthy | RCT (placebo-controlled) | - Plasma glucose concentration - Serum insulin concentration - Plasma glucagon concentration | ↑Serum insulin concentration  ↑Plasma glucagon concentration  ↔Plasma glucose concentration | ↑ Serum insulin concentration  ↑Plasma glucagon concentration  ↔Plasma glucose concentration | E&M | | | | | | | |
| **Endocrine & Metabolic + Immune + Digestive systems** | | | | | | | | | | | | | |
| Genton (2021b) [76] | Chronic haemodialysis with PEW | RCT (double-blind) | - 1-AG & 2-AG - 1-OG & 2-OG - anandamide - N-Oleoyletanolamine - N-Palmitoyethanolamine - N-Linoleoylethanolamine - N-Stearoylethanolamine - Serum IL-6 - Serum TNF-α - Serum CRP - Serum fecal IgA - Serum IL-10 (anti-inflammatory) - Gut permeability (LPS; GLP-2) - Total ghrelin - Active ghrelin - Leptin - Active GLP-1 - CCK - Neuropeptide Y - PYY - Microbiota diversity (Shannon diversity index; zOTU richness (taxa of bacterial communities)) | NA | NS (for all outcome measures) | E&M | I | | | D | | | |
| **Endocrine & Metabolic + Immune + Digestive + Muscular + Renal + Skeletal systems** | | | | | | | | | | | | | |
| Genton (2021a) [52] | Chronic haemodialysis with PEW | RCT (double-blind) | - cholesterol - parathyroid hormone - 25-OH vitamin D - Indirect calorimetry (VCO2; VO2; resting energy expenditure; respiratory quotient) - Lymphocytes - Intake and appetite (kcal; protein; appetite rating) - lean soft tissue (DXA) - lean body mass index (DXA) - fat free mass (BIA) - fat free mass index (BIA) - handgrip strength - predialysis urea - creatinine - nPCR - Kt/Vurea - bicarbonate - bone mineral content | NA | ↔cholesterol  ↔parathyroid hormone  ↔25-OH vitamin D  ↔Indirect calorimetry (VCO2; VO2; resting energy expenditure; respiratory quotient)  ↔Lymphocytes  ↔Intake and appetite (kcal; protein; appetite rating)  ↑handgrip strength  ↑fat free mass index  ↔lean soft tissue (DXA)  ↔lean body mass index (DXA)  ↔fat free mass (BIA)  ↔creatinine  ↔Kt/Vurea  ↔bicarbonate  ↑ nPCR  ↑ pre-dialysis urea  ↔ bone mineral content | E&M | I | D | | M | | R | S |
| **Endocrine & Metabolic + Nervous systems** | | | | | | | | | | | | | |
| Munts  (2009) [77] | CRPS with dystonia | RCT (double-blind, placebo-controlled) | - Plasma growth hormone concentration - Pain (numeric rating scale) - McGill pain questionnaire - Thermal sensory analyser - BFM dystonia rating scale - Unified myoclonus rating scale - Tremor research group rating scale - Radboud skills questionnaire | NS (for all outcome measures) | NR | E&M | | N | | | | | |
| **Nervous system** | | | | | | | | | | | | | |
| Bannai (2012) [28] | Healthy | RCT (single-blind, placebo-controlled) | - Sleepiness - Fatigue - Simple reaction - Psychomotor vigilance - Memory recognition - Number addition | ↓fatigue  ↓psychomotor vigilance reaction time | NR | N | | | | | | | |
| O’Neill  (2011) [32] | Healthy | RCT (double-blind, placebo-controlled) | - Sensorimotor gating by pre-pulse inhibition | ↓sensorimotor gating | NR | N | | | | | | | |
| Palmer (2008) [35] | Healthy | RCT (double-blind, placebo-controlled) | - Reaction time, accuracy (original & novel), sensitivity index measured for: delayed word; recognition; delayed picture recognition; spatial working memory; numeric working memory - Reaction time and accuracy for: digit vigilance; rapid visual information processing; choice reaction time - Simple reaction time: Critical flicker fusion (threshold) | NS (for all outcome measures) | NS (for all outcome measures) | N | | | | | | | |
| Leung  (2007) [31] | Healthy | RCT (double-blind, placebo-controlled) | ERP amplitude and latency:   - MMN (frontal and mastoid regions; laterality) - N1 (laterality; sagittality) - N2 (laterality; sagittality) - P3a (laterality; sagittality) | ↓MMN amplitude (frontal region)  ↔N1 (laterality; sagittality)  ↔N2 (laterality; sagittality)  ↔P3a (laterality; sagittality) | NR | N | | | | | | | |
| O’Neill  (2007) [34] | Healthy | RCT (double-blind, placebo-controlled) | - Auditory cortex activity (LDAEP) using EEG analysis (N1/P2 amplitude; LDAEP slope) | ↑ N1/P2 amplitude  ↓ LDAEP slope | NR | N | | | | | | | |
| Yamadera  (2007) [29] | Healthy | RCT (single-blind, placebo-controlled) | - Sleep (subjective sleep quality; subjective sleep efficiency) - PSG parameters (sleep architecture; latency to sleep stages (2, 3+4 and REM); transition of sleep phases overtime after getting into bed) - Daytime sleepiness (SSS; VAS) - Daytime cognitive function (memory recognition task; reaction time) | ↑Sleep (↑subjective sleep quality from 3 questions: Qn11 “How satisfied are you with last night’s sleep?”, “How much difficulty did you have in getting off to sleep last night?”, and Qn14 “How long did it take you to fall asleep last night?”; ↑subjective sleep efficiency)  ↓ PSG parameters (Latency to sleep onset (stage 2) and slow wave sleep (stage 3))  ↔PSG parameters (latency to REM, sleep architecture)  PSG parameters (stage 4, transition of sleep phases): NR  ↓Daytime sleepiness from VAS item 2: “How sleepy are you?”  ↑Daytime cognitive function: ↑memory recognition task (correct response ratio%) | NR | N | | | | | | | |
| Neumeister  (2006) [33] | Healthy | RCT (double-blind, placebo-controlled) | - Drowsy & emotional feelings (behavioural assessments: visual analogue scales; fatigue) - Neuropsychological function (attention/vigilance; visual; verbal and working memory; executive function; speed of processing) - Brain glucose metabolic rate: global (CMRGlu); rCMRGlu; left superior frontal gyrus; right cerebellum; left cerebellum; left transverse temporal gyrus; right hippocampus; right insula; left insula | ↓rCMRGlu (left superior frontal gyrus; right cerebellum; left cerebellum; left transverse temporal gyrus)  ↔rCMRGlu (right hippocampus, right insula, left insula)  ↔Neuropsychological function (attention/vigilance; visual; verbal and working memory; executive function; speed of processing)  ↔Drowsy & emotional feelings (behavioural assessments: visual analogue scales; fatigue) | ↓verbal & working memory  Drowsy & emotional feelings: NR  ↔Neuropsychological function (attention/vigilance; visual; executive function; speed of processing)  Brain glucose metabolic rate (global (CMRGlu); rCMRGlu; left superior frontal gyrus; right cerebellum; left cerebellum; left transverse temporal gyrus; right hippocampus; right insula; left insula): NR | N | | | | | | | |
| Inagawa (2006) [30] | Dissatisfaction with sleep | RCT (double-blind, placebo-controlled) | - Sleep - Fatigue | Sleep (↑clear-headedness from SMH Sleep Questionnaire Qn10 “How clear-headed did you feel after getting up this morning?”  ↓Fatigue from 2 items: “very lively” & “extremely peppy” | NR | N | | | | | | | |
| Heresco-Levy  (2004b) [46] | Schizophrenia | RCT (double-blind, placebo-controlled) | - BPRS - PANSS (positive symptoms; negative symptoms; cognitive symptoms; excitement; depression) - Extrapyramidal symptoms (SAS; AIMS) | PANSS 5-factor analysis (↓negative symptoms; ↓cognitive symptoms; ↓excitement; ↓depression; ↔positive symptoms)  ↓BPRS  Extrapyramidal symptoms (↓AIMS; ↔SAS) | PANSS 5-factor analysis (↓positive symptoms; ↓negative symptoms; ↓cognitive symptoms; ↓excitement; ↓depression)  Extrapyramidal symptoms (↓SAS; ↓AIMS)  ↔BPRS | N | | | | | | | |
| Heresco-Levy  (2004a) [47] | Schizophrenia | RCT (double-blind, placebo-controlled) | - PANSS (negative symptoms; positive symptoms; general psychopathology; total PANSS score) | PANSS (↓negative symptoms; ↓positive symptoms; ↓general psychopathology; ↓total PANSS score) | PANSS (↓negative symptoms; ↓positive symptoms; ↓general psychopathology; ↓total PANSS score) | N | | | | | | | |
| Javitt (2001) [45] | Schizophrenia | RCT (double-blind, placebo-controlled) | - PANSS (positive symptoms; negative symptoms; cognitive symptoms; depression symptoms; excitement symptoms) - Extrapyramidal symptoms (BAS; SAS; AIMS) | ↓ PANSS negative symptoms  ↔PANSS (positive symptoms; negative symptoms; cognitive symptoms; excitement symptoms; depression symptoms)  ↔Extrapyramidal symptoms (BAS; SAS; AIMS) | ↓PANSS (↓negative symptoms; ↓cognitive symptoms)  PANSS (positive symptoms; depression symptoms; excitement symptoms): NR  Extrapyramidal symptoms (BAS; SAS; AIMS): NR  Glycine discontinuation (up to 8 weeks after): ↓PANSS negative symptom score; ↓ PANSS cognitive symptom score | N | | | | | | | |
| Heresco-Levy  (1999) [44] | Schizophrenia | RCT (double-blind, placebo-controlled) | - PANSS (positive symptoms; negative symptoms; cognitive symptoms; excitement symptoms; depression symptoms) - BPRS - Extrapyramidal symptoms (SAS; AIMS) | PANSS (↓negative symptoms; ↓cognitive symptoms; ↓depression symptoms; ↔excitement symptoms; ↔depression symptoms)  BPRS: NR  Extrapyramidal symptoms (↔SAS; ↔AIMS) | ↓PANSS (↓negative symptoms; ↓positive symptoms  ↓cognitive symptoms; ↓depression symptoms; ↔excitement symptoms)  Glycine discontinuation (up to 8 weeks after): ↓PANSS negative symptom score  ↓BPRS  Extrapyramidal symptoms (SAS; AIMS): NR | N | | | | | | | |
| Heresco-Levy  (1996) [43] | Schizophrenia | RCT (double-blind, placebo-controlled) | - PANSS by 3-factor analysis (positive symptoms; negative symptoms; general psychopathology; total PANSS score) - PANSS by 5-factor analysis (positive symptoms; negative symptoms; cognitive symptoms; excitement symptoms; depression symptoms) - Extrapyramidal symptoms (SAS; AIMS) | PANSS 3-factor analysis (↓negative symptoms; ↓general psychopathology; ↓total PANSS score; ↔positive symptoms)  PANSS by 5-factor analysis: NR  Extrapyramidal symptoms (↔SAS; ↔AIMS) | PANSS 3-factor analysis (↓positive symptoms; ↓negative symptoms; ↓general psychopathology; ↓total PANSS score)  PANSS by 5-factor analysis (↓negative symptoms; ↓cognitive symptoms; ↓depression; ↔positive symptoms; ↔excitement symptoms)  Extrapyramidal symptoms (↔SAS; ↔AIMS) | N | | | | | | | |
| **Digestive system** | | | | | | | | | | | | | |
| Amin (2018) [78] | i) Healthy  ii) Healthy | i) RCT (double-blind, placebo-controlled*)  ii) RCT (double-blind, placebo-controlled*) | - Gut hormone release (plasma acylated ghrelin; plasma GLP-1; plasma PYY) - Subjective appetite (VAS): feelings of hunger; pleasantness to eat; prospective food intake; fullness; sickness | i) NA  ii) NA | i) NS (for all outcome measures)  ii) NS (for all outcome measures) | D | | | | | | | |
| Luiking  (1998) [79] | Healthy | RCT (double-blind, placebo-controlled*) | - Gallbladder volume - Reflux levels | NR | NR | NA | | | | | | | |
| **Muscular system** | | | | | | | | | | | | | |
| Antonio (2002) [51] | Resistance-trained | RCT (double-blind, placebo-controlled) | - Anaerobic work output measured by 2 sets each of: leg press (lower body); bench press (upper body) | NS (for all outcome measures) | NR | M | | | | | | | |
| **Non-randomised trials** | | | | | | | | | | | | | |
| **Endocrine & Metabolic systems** | | | | | | | | | | | | | |
| Kasai  (1980) [27] | Non-obese normal | Open-label trial | - Serum GH - Serum blood sugar - Serum prolactin | NA | Serum GH: ↑4g; ↔8g; ↔12g Glycine  Serum blood sugar: ↑12g; ↔8g; ↔12g Glycine  ↔Serum prolactin (all doses) | E&M | | | | | | | |
| Kasai  (1978) [26] | i) Non-obese normal; gastroduodenal anastomosis (partially gastrectomied)  ii) Non-obese normal; non-obese diabetics | Open-label trial | i & ii)   - Serum GH - Serum blood sugar - Serum prolactin (only for non-obese normal) - Serum immunoreactive insulin | NA | i) non-obese normal & gastroduodenal anastomosis populations: ↑serum GH; ↔serum blood sugar; ↔serum immunoreactive insulin  non-obese normal: ↔serum prolactin  ii) non-obese normal: ↑serum GH; ↔serum blood sugar; ↔serum prolactin, ↔serum immunoreactive insulin  non-obese diabetics: ↔serum GH; ↔serum blood sugar; ↔serum immunoreactive insulin | E&M | | | | | | | |
| **Nervous + Cardiovascular + Renal + Reproductive systems** | | | | | | | | | | | | | |
| Sugaya (2021) [48] | Overactive bladder | Pilot, double-blind, placebo-controlled | - Sleep latency - First nocturia latency - Bladder pain (VAS) - SBP - DBP - Pulse rate - Urinary frequency (diurnal; nocturnal) - Urinary, prostate & overactive bladder symptoms: OABSS (diurnal urination, nocturnal urination, urgency, incontinence, total score); N-QOL (13 items + total scores for vitality, worries, and total) - IPSS (7 items + total scores each for voiding, storage, overall) - IPSS-QOL | ↓Sleep latency  ↓Bladder pain  ↔First nocturia latency  ↔SBP  ↔DBP  ↔Pulse rate  Urinary frequency (↓nocturnal; ↔diurnal)  OABSS (↓nocturnal urination, ↓ urgency, ↓incontinence, ↓total score; ↔diurnal urination)    ↑N-QOL (Qns 1, 4, 7, vitality total, total score)  ↓IPSS (↓nocturia, ↓urgency)  ↔IPSS (incomplete emptying, frequency, intermittency, weak stream, straining, voiding total, storage total, total score)  ↔N-QOL (Q2,3,5,6,8,9,10,11,12,13; worries total)  ↓IPSS-QOL | ↓Sleep latency  ↓First nocturia latency  ↓Bladder pain  ↓SBP  ↓DBP  ↔Pulse rate  Urinary frequency (↓nocturnal; ↔diurnal)  OABSS (↓nocturnal urination, ↓total score; ↔diurnal urination; ↔urgency; ↔incontinence)  ↑N-QOL (Qns 1, 3, 5, 6, 7, 10, 12, vitality total, worries total, total score)  ↔N-QOL (Q2,4,8,9,11,13)  IPSS (↓nocturia, ↓storage total)  ↔IPSS (incomplete emptying, frequency, intermittency, weak stream, straining, voiding total, urgency, total score)  ↓IPSS-QOL | N | C | | | R | | Rp | |
| **Nervous system** | | | | | | | | | | | | | |
| Truong (1988) [80] | Myoclonus | Open-label trial; Double-blind, placebo-controlled (crossover) | - Severity & quality of myoclonus (motor scores; functional scores; global assessment) | NS (for all outcome measures) | NS (for all outcome measures) | N | | | | | | | |
| Strzelecki  (2011) [49] | Schizophrenia | Open-label trial | - PANSS (positive symptoms; negative symptoms; general psychopathology; total PANSS score) - Cognition (working memory; executive functions) | NA | PANSS: ↓positive symptoms, ↓negative symptoms, ↓general psychopathology symptoms, ↓total PANSS score  Cognition: ↑working memory, ↑executive functions | N | | | | | | | |
| Javitt (1994) [42] | Schizophrenia | Open-label trial | - PANSS (negative symptoms, positive symptoms, general psychopathology) | NA | PANSS: ↓negative symptoms, ↔positive symptoms, ↔general psychopathology | N | | | | | | | |
| Rosse (1989) [81] | Chronic psychotic disorder | Open-label, pilot trial | - Psychiatric (BPRS; SANS; CGI scale) - Cognition (memory) - Extrapyramidal symptoms (SAS; AIMS) | NA | NS (for all outcome measures) | N | | | | | | | |

Overall outcome (for each physiological system): green = positive effect, red = negative effect, blue = miscellaneous effect (includes positive &/or inconclusive effects), yellow = no statistically significant effect

(↑) = statistical significant increase of p < 0.05, (↓) = statistical significant decrease of p < 0.05, (↔) = no statistical significant change of p < 0.05, (*) = glycine as placebo, (^#^) = % participants who withdraw or drop-out of study, (♀) = female, (**♂)** = male, (X) = crossover trial, (//) = parallel trial, A1C = glycated hemoglobin, AG = arachidonoylglycerol, AIDS = acquired immune deficiency syndrome, AIMS = abnormal involuntary movement scale, ARV = antiretroviral, BAS = Barnes Akathisia Scale, BIA = bioelectrical impedance analysis, BMI = body mass index, BPRS = brief psychiatric rating scale, C = cardiovascular, CAD = coronary artery disease, CCK = cholecystokinin, CD = cluster of differentiation, CDRS = clinical dementia rating scale, CGI = clinical global impression, CPK = creatine phosphokinase, CRP = C-reactive protein, CDAI = Crohn’s disease activity index, Cr-EDTA = chromium ethylenediamine tetraacetic acid, CSF = cerebrospinal fluid, D = digestive, DBP = diastolic blood pressure, DXA = dual-energy X-ray absorptiometry, E&M = endocrine & metabolic, ECG = electrocardiogram, ERP = event-related potential, GI = gastrointestinal, I = immune, IFN-γ = interferon gamma, IGF1 = insulin-like growth factor 1, IgA = immunoglobulin A, IgG = immunoglobulin G, IgM = immunoglobulin M, IL-; interleukin, IL-1β, interleukin-1 beta, IPSS-QOL = international prostate symptom score- quality of life, GABA = gamma-aminobutyric acid, GH = growth hormone, GLP-1 = glucagon-like peptide-1, GLP-2 = glucagon-like peptide-2, HDL = high-density lipoprotein, HIV = human immunodeficiency virus, HOMA-IR = homeostatic model assessment for insulin resistance, LAD = left anterior descending artery, LCX = left circumflex artery, LDAEP = loudness dependence of auditory evoked potentials, LDL = low-density lipoprotein, LPS = lipopolysaccharides, LM = left main stem, LTR = lymphocyte transformation ratio, M = muscular, MMN = mismatch negativity, N = nervous, N-QOL = nocturia-quality of life, NA = not applicable, nPCR = normalised protein catabolic rate, NPI = neutrophil phagocytosis index, NMDA = N-methyl-D-aspartate, NR = not reported, NS = non-significant, OABSS = overactive bladder symptom score, OBS = obsessive, OCDS = Obsessive Compulsive Drinking Scale, OG = oleoylglycerol, OSS = Orgogozo Stroke Scale, PAI-2 = plasminogen activator inhibitor-2, PANSS = positive and negative syndrome scale, PEW = protein-energy wasting, PSG = polysomnography, PYY = peptide tyrosine tyrosine, R = renal, RBC = red blood cell, RCA = right coronary artery, rCMRGlu = regional cerebral glucose metabolic rate, RCT = randomised controlled trial, REM = rapid eye movement, Rp = reproductive, S = skeletal, SANS = scale for the assessment of negative symptoms, SAS = Simpson Angus Scale, SBP = systolic blood pressure, SMH = St Mary’s Hospital, SSS = Stanford Sleepiness Scale, T2DM = type 2 diabetes mellitus, TBARS = thiobarbituric acid reactive substances, TBSA = total body surface area, TC = total cholesterol, TG = triacylglycerols, TNF-RI = tumour necrosis factor receptor 1, VAS = visual analog scale, VCO2 = volume of carbon dioxide, VO2; volume of oxygen, WSAS = work and social adjustment scale, WTAR = Wechsler Test of Adult Reading, Y-BOCS = Yale-Brown obsessive compulsive scale
